# Emerging regenerative strategies for spinal cord injury: exosome-derived mechanisms and therapeutic insights

**DOI:** 10.3389/fnins.2025.1652196

**Published:** 2025-08-25

**Authors:** Haixia Fan, Jie Gao, Qian Chen, Shuangshuang Sun, Jinshen Guo, Xiaodong Liu, Jinhu Li

**Affiliations:** ^1^First Hospital of Shanxi Medical University, Taiyuan, Shanxi, China; ^2^Academy of Medical Sciences, Shanxi Medical University, Taiyuan, Shanxi, China

**Keywords:** spinal cord injury, exosomes, inflammation, neuroprotection, mesenchymal stem cells

## Abstract

**Background:**

Spinal cord injury (SCI) often leads to severe motor and sensory impairments, and current treatment methods have not achieved complete neural repair. In recent years, exosomes have become a research focus in the treatment of nerve injuries due to their important roles in intercellular information transfer, immune regulation, and neural repair. Our study conducts a scientometric analysis to map the research landscape related to exosomes in SCI.

**Methods:**

Articles and reviews related to exosome in SCI were retrieved from the Web of Science Core Collection and Scopus. Analysis was conducted using Microsoft Excel 2021, CiteSpace (6.4.R1), VOSviewer (1.6.18), the R software (4.4.3) bibliometrix package, etc.,

**Results:**

Since 2018, the number of publications has rapidly increased. Fan Jin is the most academically influential author in the field, while Cai Weihua’s research has received widespread recognition from researchers. China is the leading contributor among the 32 countries/regions. Among the 708 institutions, Central South University and Zhejiang University are the primary supporters. *Journal of Nanobiotechnology* is the most influential journal in this field, with *Neural Regeneration Research* and *Cells* also making significant contributions. Keyword analysis focuses on “mesenchymal stem cells,” “inflammation,” “cell therapy,” “axonal regeneration,” “functional recovery,” “neuroinflammation,” “neurodegeneration,” “ferroptosis,” “pyroptosis,” and “precision medicine” emphasizing cellular therapies for tissue repair. Emerging topics like “nanoparticles” show significant potential in SCI treatment, further enhancing regenerative medicine approaches.

**Conclusion:**

Our study show that the growing global interest in exosome-based therapies for SCI, marking an important step in understanding their preclinical potential. These therapies show promise in promoting neuroprotection, axonal regeneration, and modulating inflammation. Moving forward, future research will focus on further exploring the integration of exosome therapies with advanced drug delivery systems and regenerative medicine, aiming to enhance SCI treatments and tailor recovery strategies in preclinical models.

## 1 Introduction

The incidence and prevalence of Spinal cord injury (SCI) have significantly increased globally, becoming a serious medical and social burden (Lu et al., 2025). Currently, the treatment methods for SCI mainly include acute phase treatment, surgical treatment, medication, and rehabilitation. Acute phase treatment relies on steroids to reduce inflammation (Liu et al., 2019), surgical treatment alleviates spinal cord pressure through decompression and stabilization (Bagnall et al., 2008; Maas et al., 2021), medication uses neuroprotective drugs and antispasmodics to relieve inflammation and spasms (de Sousa et al., 2022; Fehlings et al., 2023), and rehabilitation improves function through physical therapy and neuromuscular electrical stimulation (Harvey, 2016; Yang et al., 2022). However, these treatment methods have limited effectiveness, and recovery from SCI still faces significant challenges.

Exosomes are a type of nanoscale membrane vesicle secreted by various cells (such as immune cells, stem cells, tumor cells, etc.), with a diameter typically ranging from 40 to 100 nm. They are widely present in body fluids such as blood, urine, and breast milk (Simpson et al., 2008). Exosomes play a pivotal role in intercellular communication by facilitating the transfer of bioactive molecules, including proteins, lipids, mRNA, and miRNA, to recipient cells (Fang W. et al., 2025). This intercellular trafficking regulates immune responses, promotes tissue repair and regeneration, and is implicated in a wide range of physiological and pathological processes (Wang Y. et al., 2025; Yang W. et al., 2025). Recent studies have emphasized the therapeutic potential of exosomes in modulating these processes, making them a focal point of biomedical research (Simons and Raposo, 2009). Exosomes have shown significant therapeutic potential in multiple fields, including neurodegenerative disease (Rehman et al., 2023; Tao and Gao, 2024), cardiovascular diseases (Zhang et al., 2024), tumors (Hu et al., 2023; Wang T. et al., 2025; Yang et al., 2024), diabetic complications (Jiao et al., 2024) and immune-related diseases (Gangadaran et al., 2023). Exosomes, as an emerging treatment method for SCI, show significant potential. Research indicates that exosomes exert various functions such as anti-inflammatory (Singh et al., 2024), anti-apoptotic (McMullan et al., 2025), promoting axonal regeneration (Dong et al., 2025), angiogenesis (Zhai et al., 2025) and myelin repair (Chai et al., 2024) by delivering active factors like miRNA and proteins (Kim et al., 2024). However, the role of exosomes in SCI is not solely therapeutic. Research also indicates that exosomes play a crucial role as “pathological signal carriers” in the SCI pathology (Singh et al., 2025a). In conclusion, the role of exosomes in SCI treatment is complex and bidirectional: on one hand, they promote neural recovery by delivering active factors; on the other hand, in the pathological context, they may contribute to the formation of glial scars, thereby inhibiting neurogenesis. Therefore, precise control of exosome release and its effects will be an important research direction for the future treatment of SCI.

Bibliometric analysis is a method of quantitatively analyzing information in a specific field, using visualization and network technologies to reveal research trends in that field. Through bibliometric analysis, researchers can quickly understand the current state of research and predict future research hotspots (Cooper, 2015). Scientometric analysis, on the other hand, focuses on the cutting-edge dynamics and development trends of specific fields or disciplines, aiming to explore in depth the scientific research progress on a particular topic, the broader situation in related fields, and even the entire scientific knowledge system (Chen et al., 2012; William et al., 2001). The role of exosomes in the treatment of SCI is a complex and critical research area, but there is currently a lack of bibliometric and scientometric analysis on this topic.

To bridge the gap, we conducted a comprehensive bibliometric analysis of the literature on exosomes and SCI over the past 15 years, systematically mapping the intellectual landscape, research frontiers, and emerging trends in the field. The goal of this study is to provide a scientific basis for the treatment and rehabilitation of SCI through an in-depth analysis of the role of exosomes.

## 2 Materials and methods

### 2.1 Data source

The data for this study were retrieved on 5 June 2025, using the Web of Science Core Collection (WoSCC) and Scopus databases. WoSCC search strategy: [TS = spinal cord injury (Mesh)] AND [TS = (“exosome*” OR “exosomal”)], and Scopus search strategy: [(spinal cord injury (MeSH)] AND [“exosome*” OR “exosomal”)]. Duplicate references were removed using R and Excel, and two types of articles were selected: original research articles and reviews. A total of 768 relevant papers were retrieved in this search. All documents were in English. Additionally, a literature search was conducted in the PubMed databases (Supplementary Figure 1). According to the exclusion criteria, we included only “clinical trial” articles and selected only those published in English. However, the search results showed that only one article related to clinical trials met our inclusion criteria.

In this study, we focused on literature related to exosomes, and our search terms only included the term “exosomes.” We did not use the broader term “extracellular vesicles” because our research is specifically focused on the concept of exosomes. The term “extracellular vesicles” encompasses a variety of vesicle types, including exosomes and microvesicles, which could result in an overly broad search scope.

### 2.2 Data processing

Data extracted from the Scopus database is converted into a plain text file format compatible with the Web of Science (WOS) database using CiteSpace (6.4.R1). The bibliometric analysis is then conducted on the plain text files from both the Scopus and WOS databases, employing CiteSpace, the bibliometrix package in R (4.4.3), and VOSviewer (1.6.18). A circular Sankey diagram is created using Charticulator to visualize the results of the analysis, providing a clear representation of the relationships and trends identified in the data.

### 2.3 Data analysis

**FIGURE 1:**
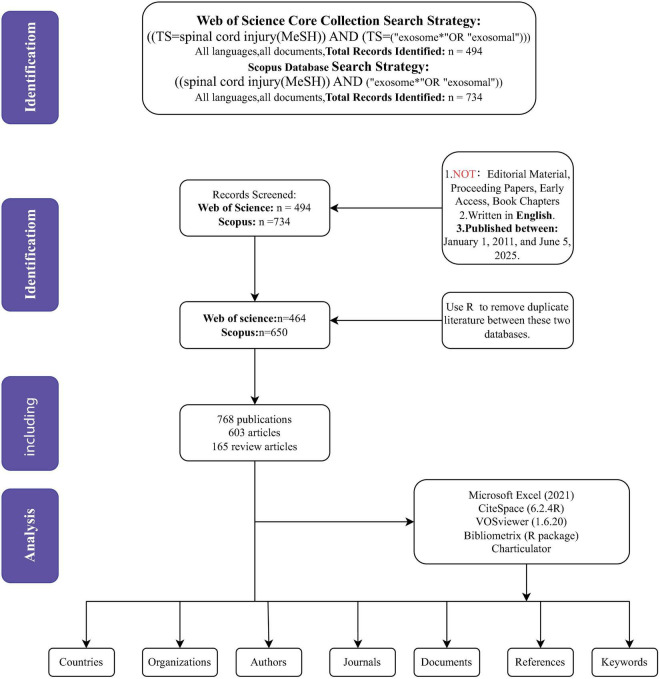
The flowchart for literature search, selection and analysis. TI, title, AK, author keywords, AB, abstract.

Multiple tools were used to analyze and verify the data: CiteSpace was employed for co-occurrence analysis, clustering, and emergent analysis; VOSviewer was used for co-occurrence analysis and clustering; the bibliometrix package was used for frequency analysis, relational network analysis. Additionally, the journal names, impact factors (IF), and journal rankings (Q1–Q4) were recorded using the 2021 edition of the Journal Citation Reports (JCR). Excel was used to create bar charts, line charts, and stacked area charts. Due to differences in the distribution of countries, institutions, journals, and authors across various fields, and because CiteSpace primarily reviews literature from the past 5 years, the results from the four tools may differ slightly. In such cases, the results from the bibliometrix package were prioritized when discrepancies arose between the tools. The data quality was verified to be acceptable, and subsequent analysis was carried out based on these verified results (Figure 1).

### 2.4 Terminology clarification

The terms “mesenchymal stromal cells” and “mesenchymal stem cells” are used interchangeably. While these two terms may have nuanced differences in certain contexts, they are often used synonymously in the literature, especially in studies related to regenerative medicine and exosome therapies. For the purpose of this research, both terms refer to multipotent cells derived from mesenchymal tissues with regenerative potential, and are treated as equivalent when discussing their role in exosome-based therapies for spinal cord injury. This approach ensures consistency in the analysis and simplifies the presentation of the findings without introducing unnecessary complexity.

## 3 Results

### 3.1 Study identification and characteristics

**FIGURE 2:**
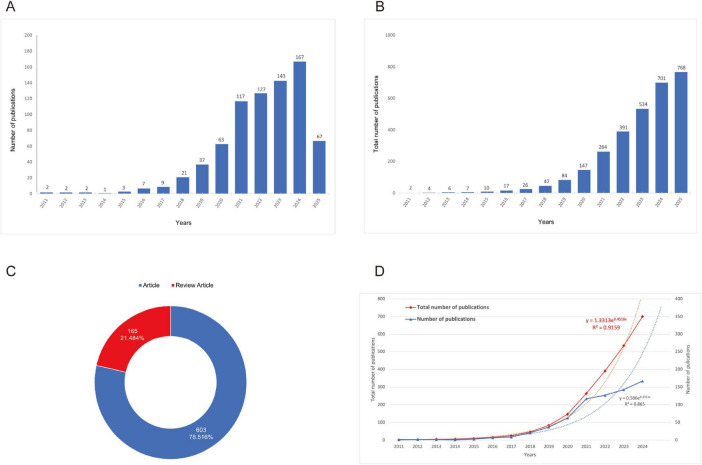
Growth trends and distribution of publications. **(A)** Growth trend of publications (the purple bars represent the number of publications, while the blue line indicates the number of citations.); **(B)** Cumulative growth trend of publications (the data reveals a steady increase in publications over the years, with a particularly sharp rise starting in the early 2018’s.); **(C)** Proportion of research articles versus review articles [the blue segment represents the articles (78.516%), and the red segment represents the review articles (21.484%.)]; **(D)** Comparison of total and annual number of publications (the red line shows the total number of publications, and the blue line represents the annual publications. The regression equations and R^2^ values indicate strong exponential growth in both metrics.)

A total of 768 publications were identified and included in the bibliometric analysis. The growth trend of publications (Figures 2A, B) illustrates a steady upward trend in annual publication volume commencing in 2011, with a notably accelerated growth rate observed after 2018. Publication output reached its peak in 2024, reflecting sustained and increasing academic interest in this research domain. Regarding publication type (Figure 2C), original research articles constituted the majority (78.5%, *n* = 603), while review articles represented 21.5% (*n* = 165). Regression analysis confirmed the exponential growth pattern of annual and total number of publications. The total number of publications exhibits a high determination coefficient (R^2^ = 0.9159), further emphasizing the accelerated development of the literature (Figure 2D).

Bibliometric analysis revealed dynamic growth in spinal cord injury exosome research from 2011 to 2025, exhibiting a 28.51% mean annual growth rate with peak productivity in 2024 (Supplementary Table 1). The corpus demonstrated strong scholarly impact, reflected by a mean citation rate of 33.74 per document and 53,244 collectively cited references, while maintaining a recent publication profile (mean document age: 2.92 years). Collaboration patterns showed clear dominance of multi-authored works (total authors = 3,586; single-author publications: negligible), with 8.07% involving international co-authorship. These metrics confirm rapidly expanding global scholarship and intensive research collaboration in the field (Supplementary Table 1).

### 3.2 Analysis of countries/regions and institutions

**FIGURE 3:**
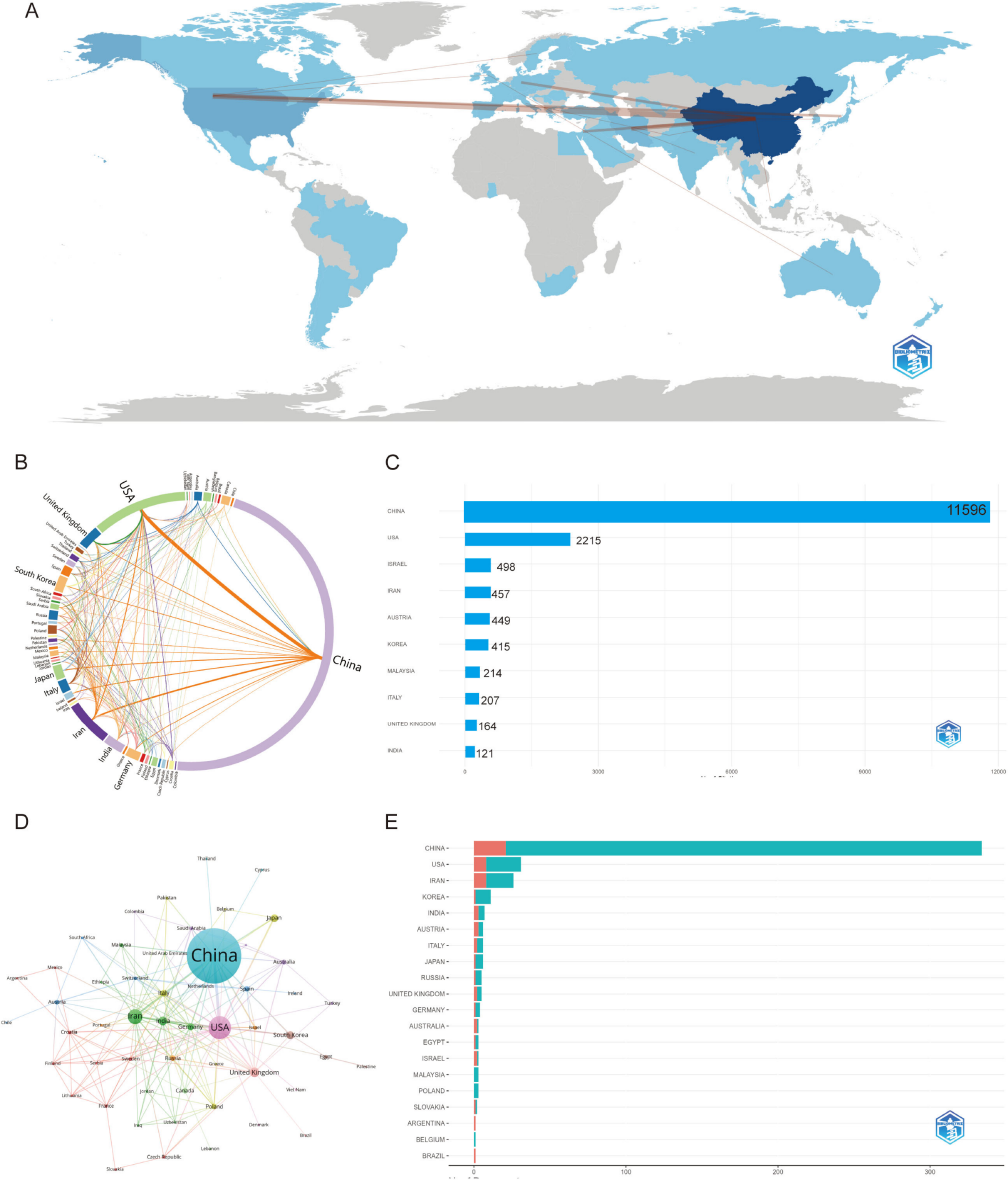
Global trends and collaboration networks in scientific publications on exosomes in spinal cord injury (2011–2025). **(A)** Global collaboration map of co-authorship networks (Bibliometrix) (darker blue shades reflect higher publication output, with lines depicting international collaborative ties.); **(B)** Country collaboration network (Charticulator) (this diagram shows how countries work together on research. Each curved line represents joint publications between two countries. Thicker lines mean more cooperation). **(C)** Country influence by citation impact (Bibliometrix) (this chart illustrates the citation impact by country, highlighting the most influential nations in the research field.); **(D)** Country collaboration network (Bibliometrix) (this network map shows the connections between countries based on their co-authored research papers. Each circle represents a country — larger circles mean more publications. Lines between countries show collaboration, and the closer the countries are, the stronger their research ties). **(E)** The top 10 countries responsible for the number of studies (this chart shows the countries with the highest number of published studies, ranked accordingly). MCP, multiple-country publications; SCP, single-country publications.

National bibliometric analysis demonstrates China’s dominance in both publication output (predominantly Single-Country Publications/SCP; Figure 3E) and total citation impact (TC = 11,596; Figure 3C), with the United States ranking second in production volume while exhibiting a higher Multi-Country Publication (MCP) ratio. Emerging research economies—including Iran, South Korea, and India—show increasing MCP engagement despite moderate output, whereas developed nations (e.g., United Kingdom, Germany) maintain characteristically high MCP levels. Citation distribution reveals Israel’s disproportionate global influence (TC = 498) and specialized impact from Austria, South Korea, and Malaysia (Supplementary Table 2). Co-authorship network visualizations (World Map, Chord Diagram, Network Analysis; Figures 3A, B, D) confirm China’s pivotal role in global scientific collaboration, demonstrating robust partnerships with the United States and European hubs that collectively drive transformative cross-border scholarship. China’s academic influence is significant, with a high total citation count, but its average citations per article are relatively low. This suggests that while China produces a large volume of research, there is room for improvement in terms of quality, and greater global collaboration is needed. In contrast, the academic systems in the United States and European countries are well-established, and although their total citation counts are lower, their research tends to have a higher impact, reflecting high-quality scholarly output. Israel and Austria, by focusing on specialized fields, have achieved disproportionately high global influence (Table 1). Therefore, the difference in average citations between China and Israel may reflect not only a difference in quality but also differences in academic publishing practices, international collaboration, and field-specific citation norms.

**TABLE 1:** Top 10 countries by total citation count in academic articles (2011–2025).

Country	Total citations	Average article citations
China	11,596	34.70
United States	2,215	71.50
Israel	498	166.00
Iran	457	17.60
Austria	449	74.80
Korea	415	37.70
Malaysia	214	71.30
Italy	207	34.50
United Kingdom	164	32.80
India	121	17.30

**FIGURE 4:**
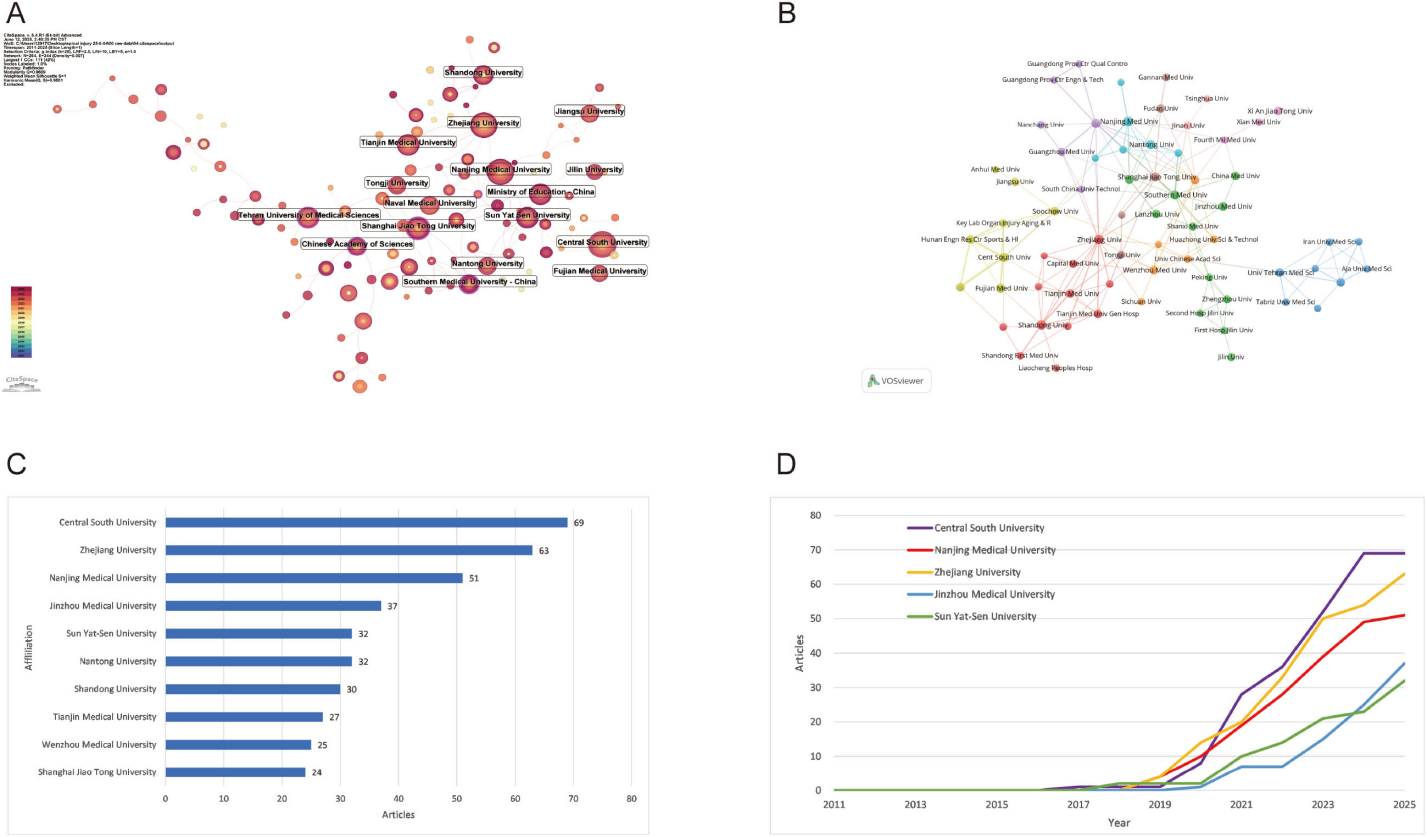
Institutional contributions and collaboration networks on exosomes in spinal cord injury. **(A)** Institution collaboration network (CiteSpace) (this diagram shows the research collaboration between institutions. The size of each node reflects the number of published articles, and the thickness of the lines indicates the strength of their connections.); **(B)** Institution co-occurrence network (VOSviewer) (each node represents an institution, and the thickness of the lines indicates the strength of their connections. Institutions are grouped by color to highlight collaboration clusters.); **(C)** Top 10 institutions by number of publications (excel); **(D)** Research publication trends of the top 5 institutions (excel).

Bibliometrix analysis identified 708 institutions across 32 countries/regions. Central South University led institutional output (69 publications), demonstrating substantial post-2018 growth that reflects its rising academic prominence (Figures 4C, D). Zhejiang University ranked second (63 publications), exhibiting a synchronized acceleration trajectory indicative of enhanced research productivity. Nanjing Medical University contributed 51 publications with analogous expansion patterns, signifying institutional prioritization of scholarly output. Collaboration network mapping (Figures 4A, B) confirmed these institutions’ centrality within global research networks, establishing them as key hubs fostering transformative academic partnerships.

### 3.3 Analysis of authors and co-cited authors

**FIGURE 5:**
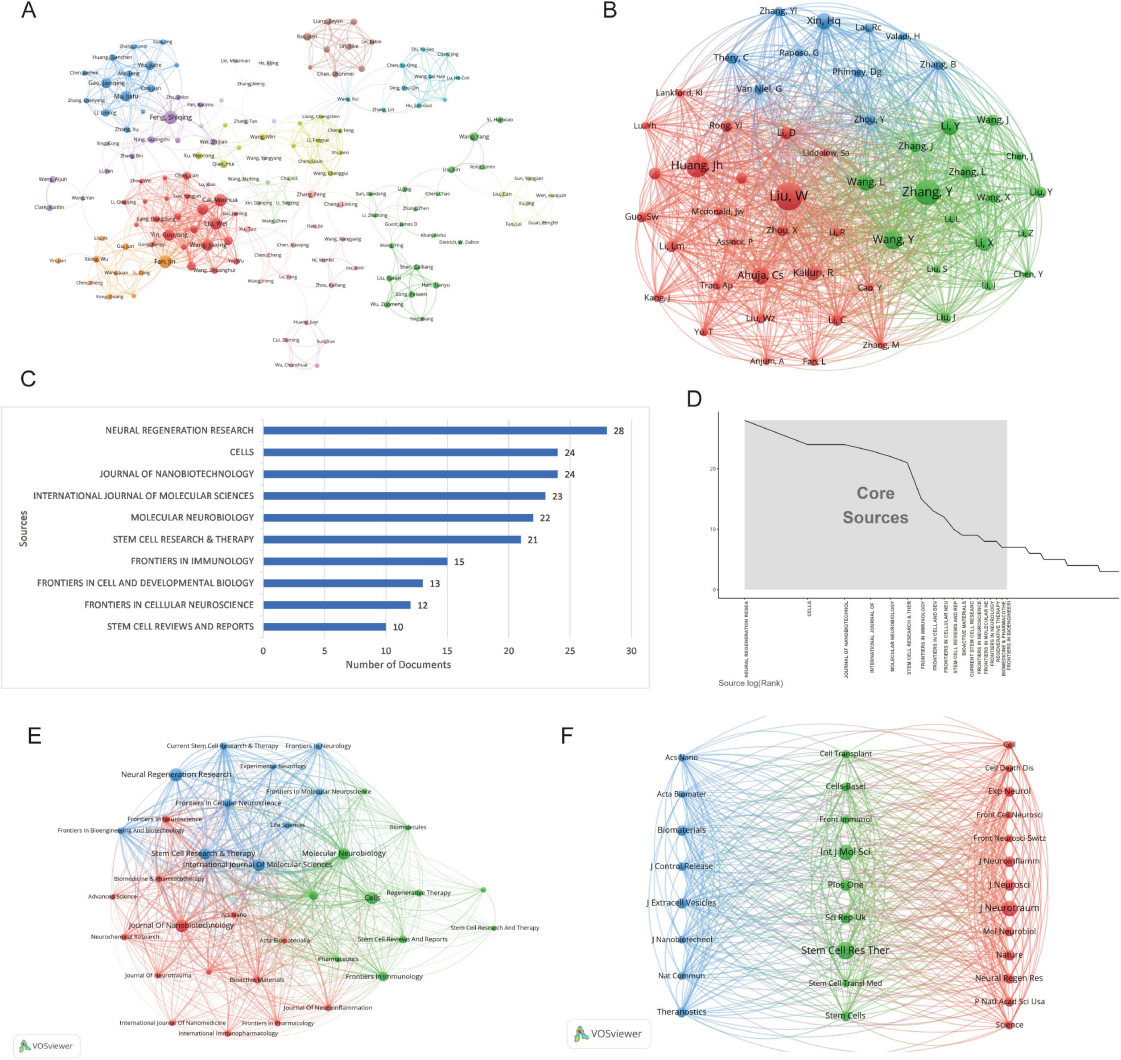
Network analysis of authors and journals on exosomes in spinal cord injury: co-authorship and co-citation relationships. **(A)** Co-authorship network map (the network graph represents the collaboration patterns among research institutions or authors. Each node corresponds to an institution or author, with the size of the node indicating the degree of collaboration and the colors representing different research fields or regions.); **(B)** Co-cited author network map (this network illustrates the relationships between authors based on co-citation patterns. The colors represent different clusters of authors, with the size of the nodes indicating the frequency of co-citations.); **(C)** Top 10 journals by number of publications (excel) (the length of each bar represents the publication count, highlighting the journals with the most published research articles). **(D)** Core journal identification based on Bradford’s law (Bibliometrix) (the x-axis shows the rank of journals based on the number of articles published, while the y-axis indicates the number of articles.); **(E)** Journal co-occurrence network map (VOSviewer) (each node represents a journal, with the size of the node indicating the number of publications. The colors represent different research areas, and the lines connecting the nodes reflect the strength of the connections between journals based on shared topics.) **(F)** Co-cited journal network map (VOSviewer) (each node represents a journal, with the size of the node reflecting the frequency of co-citations. The colors distinguish different clusters of journals, and the lines connecting the nodes represent the strength of the co-citation relationships between them).

Co-authorship network map (Figure 5A) illustrates the author collaboration network derived from bibliometric analysis, revealing systematic patterns of scholarly cooperation and academic influence within the field. The network comprises 14 distinct clusters, with the core collaborative group centered around Fan Jin (33 links; total link strength: 131), Liu Wei, and Cai Weihua. This group demonstrates extensive collaborative reach and high scholarly output (> 12 publications; > 1,500 collective citations). High-impact authors include Dietrich W. Dalton (94 citations per paper) and Chen Jiachen (171 citations per paper), both exhibiting exceptional normalized citation impact (peak value: 19.47), underscoring their pivotal contributions. Emerging researchers like Guest James D. (mean publication year: 2025), while currently less prolific, show significant potential. The network topology further reveals divergent research teams, highlighting both tightly-knit large-scale collaborations and relatively independent subgroups. These structural insights elucidate knowledge production dynamics, core research forces, and evolving scholarly trends in the field.

The co-cited author network map (Figure 5B) reveals three distinct research clusters, each with its characteristic collaboration patterns. The red cluster, anchored by Huang, Jh (citation count: 238) and Liu, W (total link strength: 3,937), demonstrates the strongest connectivity and citation impact, indicating their leadership in experimental neuroscience research. The green cluster, featuring Zhang, Y (citation count: 283) and Wang, Y (total link strength: 2,153), showcases robust interdisciplinary connections, suggesting a focus on translational research applications. Meanwhile, the blue cluster is characterized by more focused collaboration among theoretical researchers, including Xin, Hq (citation count: 164) and Phinney, Dg. Network topology analysis identifies Zhang, Y and Liu, W as pivotal hubs bridging different research domains. Furthermore, the geographical distribution of authors highlights both regional (primarily Chinese researchers in the green cluster) and international (notably, Théry, C and Van Niel, G in the red and blue clusters) collaboration patterns. These structural insights provide valuable perspectives on the current research landscape and knowledge diffusion pathways in this field.

### 3.4 Analysis of journals and co-journals

Based on the bibliometric analysis using the bibliometrix package, a total of 768 papers are distributed across 308 journals. The journal with the highest number of related publications is Neural Regeneration Research (28 papers, 3.65%), followed by Cells (24 papers, 3.13%), Journal of Nanobiotechnology (24 papers, 3.13%), International Journal of Molecular Sciences (23 papers), and Molecular Neurobiology (22 papers) (Figures 5C and Table 2). In terms of journal impact, as measured by the h-index, Journal of Nanobiotechnology holds the highest impact among the 308 journals in this field, with an h-index of 15. The top five journals with the highest h-indices are Cells (h-index = 14), International Journal of Molecular Sciences (h-index = 14), Stem Cell Research & Therapy (h-index = 12), and Neural Regeneration Research (h-index = 11) (Table 2).

**TABLE 2:** Top 10 influential journals on exosomes in spinal cord injury research (2011–2025).

Rank	Source	h_index	g_index	m_index	TC	NP	PY_start	IF	JCR
1	Journal of Nanobiotechnology	15	24	2.5	923	24	2020	10.6	Q1
2	Cells	14	24	2	1092	24	2019	5.1	Q2
3	International Journal of Molecular Sciences	14	23	1.556	796	23	2017	4.9	Q1
4	Stem Cell Research and Therapy	12	21	1.5	831	21	2018	1.2	Q4
5	Frontiers in Immunology	11	15	1.571	509	15	2019	5.7	Q1
6	Neural Regeneration Research	11	21	1.833	487	28	2020	5.9	Q1
7	Frontiers in Cell and Developmental Biology	9	13	1.5	325	13	2020	4.6	Q1
8	Frontiers in Cellular Neuroscience	9	12	1.286	249	12	2019	4.2	Q2
9	Molecular Neurobiology	9	18	1.125	348	22	2018	4.6	Q1
10	Frontiers in Molecular Neuroscience	7	8	0.875	189	8	2018	3.5	Q2

IF, impact factor; JCR, Journal Citation Reports.

Bradford’s Law is a fundamental principle in bibliometrics, suggesting that scientific journals in a given field can be divided into core and subsequent sectors based on the number of publications, following a 1:n:n^2^ distribution. In this study, we analyzed 18 core journals (Figure 5D). Among the journals in the core field, as defined by Bradford’s Law, and the top five journals by publication count, more than half are categorized as JCR Q1 journals. This indicates a high quality of the literature included in this analysis (Table 2). The journals with the highest impact factors are *Journal of Nanobiotechnology* (IF: 10.6, Q1) and *Neural Regeneration Research* (IF: 5.9, Q1).

Using VOSviewer, we constructed collaboration and co-citation journal networks and conducted classification (Figures 5E, F). The analysis of journals with more than five publications revealed that the network comprises 35 core journals that meet the criteria, forming three distinct clusters with characteristic disciplinary focuses (Figure 5E). The first cluster (blue) centers around Stem Cell Research and Therapy, focusing primarily on stem cell clinical translation research. The second cluster (red) includes journals such as Journal of Nanobiotechnology, which concentrate on nanomaterials and tissue engineering research. The third cluster (green) is represented by Frontiers in Immunology, with an emphasis on exploring the mechanisms of neural regeneration. Remarkably, the collaboration network among these high-output journals exhibits a significant multi-center structural pattern. Stem Cell Research & Therapy serves as the core hub of the network (degree centrality = 32), maintaining close collaborations with several secondary journals (average collaboration strength = 7.8). Cross-cluster analysis shows that immunology journals play a critical bridging role in linking different research directions, while emerging high-output journals in recent years tend to form interdisciplinary collaborations, accounting for 63% of new collaborations.

The co-citation network (co-citations ≥ 260) includes 30 high-impact journals (Figure 5F), which can be categorized into three primary clusters. The first cluster (blue) focuses on nanotechnology and biomaterials, with journals such as *Acs Nano* and *Biomaterials*, reflecting the rapid advancement of nanomedicine. The second cluster (green) is centered on stem cell and immunology research, with *Stem Cell Res Ther* standing out for its high total link strength and citation count, highlighting its pivotal role in interdisciplinary research. The third cluster (red) includes top-tier journals like *Cell*, *Nature*, and *Science*, alongside specialized journals in neuroscience, such as *J Neurosci* and *J Neurotraum*, underscoring the close integration of basic research and clinical applications. Notably, open-access journals, such as *Plos One* and *Sci Rep*, hold an equal standing within the network alongside traditional high-impact journals, illustrating the widespread recognition of open science. The analysis of co-citation relationships reveals clear knowledge flow patterns, including the translation of nanotechnology into biomedical applications and the deep intersection between stem cell research and immunology.

### 3.5 Analysis of references and articles

**FIGURE 6:**
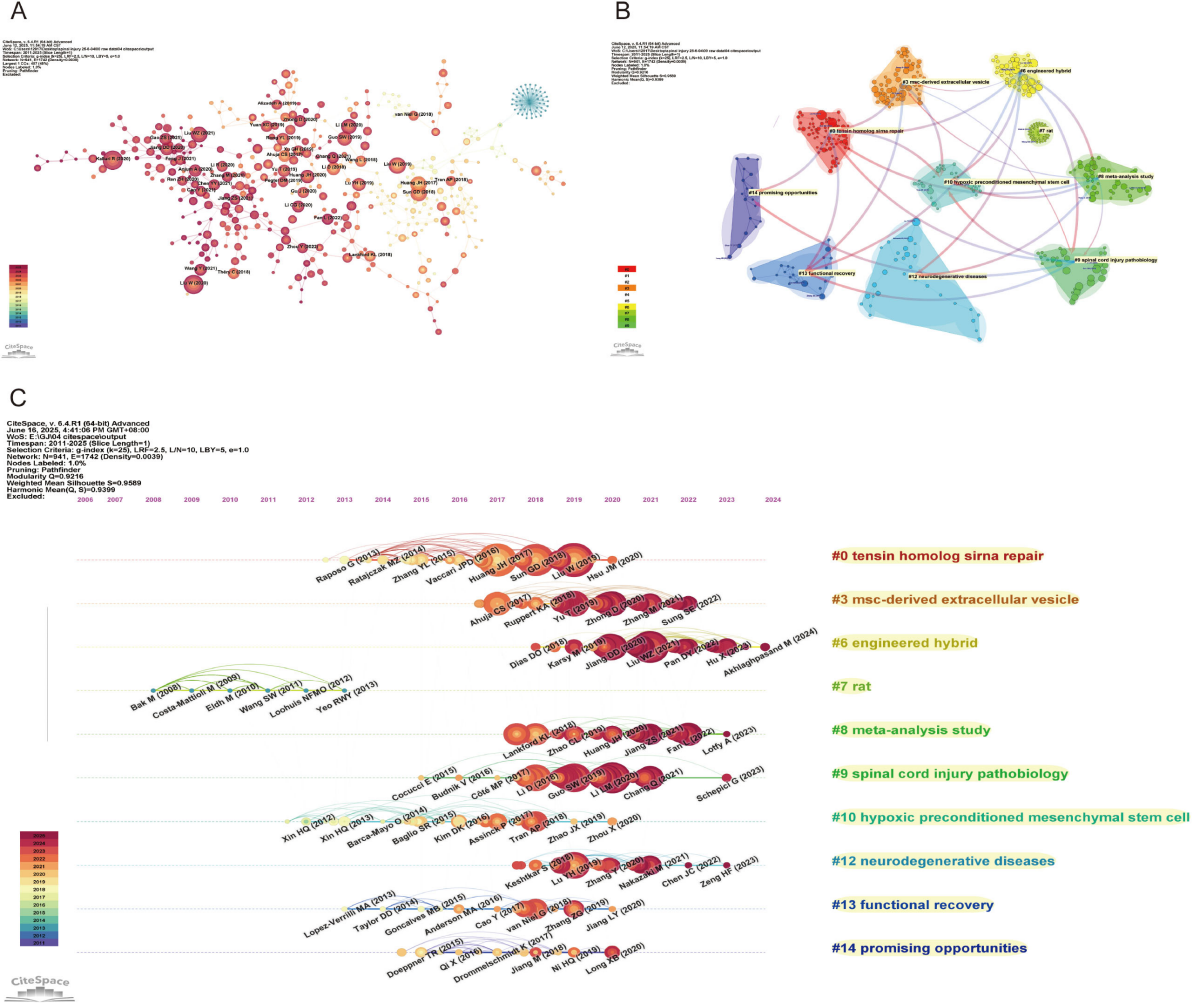
Network Visualization of references on exosomes in spinal cord injury. **(A)** Reference co-citation network map (CiteSpace) (each node represents a reference, with the size of the node indicating its influence. The lines between the nodes represent the connections between references, showing their relationships based on co-citation. The color gradient reflects the publication years, with newer references in darker shades.); **(B)** Reference clustering dependency map (CiteSpace) (each color represents a different cluster, and the lines indicate the dependency relationships between the clusters.); **(C)** Reference clustering timeline map (CiteSpace) (each node represents a reference, with the color intensity increasing as the reference approaches the present time. Horizontal lines indicate the clustering of references).

The reference collaboration network (Figure 6A) shows that core references such as Kalluri and LeBleu (2020), Li et al. (2020), Zhou et al. (2022) are located at the center of the network, with numerous collaboration links and citations. Their research has garnered significant attention in recent years, resulting in larger nodes and strong academic connections with other researchers.

The CiteSpace-generated cluster dependency diagram and cluster timeline diagram provide an in-depth analysis of the citation network, revealing the citation relationships between different literature themes and their temporal trends. The cluster dependency diagram (Figure 6B) illustrates the relationships between various research fields based on citations, displaying a total of ten clusters: “Tensin homolog siRNA repair’ (#0) focuses on gene repair and RNA interference techniques, especially research related to tensin homolog, offering new insights into cellular repair and gene therapy; “MSC-derived extracellular vesicles” (#3) mainly involves research on extracellular vesicles derived from mesenchymal stem cells (MSCs), exploring their applications in medicine and stem cell therapies; “Engineered hybrid” (#6) represents interdisciplinary innovative research, particularly in the field combining materials science and biology, emphasizing the application of engineered hybrids; “Rat” (#7) is dedicated to research using rodent models, particularly for neuroscience and disease models, providing essential experimental data to support human disease treatment; “Meta-analysis study” (#8) focuses on meta-analytic research, aiming to integrate multiple study results and assess relationships and differences between various studies; “Spinal cord injury pathobiology” (#9) addresses the pathophysiology of spinal cord injuries, exploring biological mechanisms and therapeutic strategies for spinal cord damage; “Hypoxic preconditioned mesenchymal stem cells” (#10) investigates the effects of hypoxic preconditioning on mesenchymal stem cells and their applications in regenerative medicine, advancing innovation in stem cell therapies; “Neurodegenerative diseases” (#12) deals with neurodegenerative diseases, such as Alzheimer’s and Parkinson’s disease, studying their molecular mechanisms and exploring potential treatments; “Functional recovery” (#13) focuses on functional recovery, particularly in the rehabilitation of nerve and muscle damage, dedicated to improving patients’ recovery processes; “Promising opportunities” (#14) represents emerging research fields with significant potential for development, reflecting innovative and promising themes in current scientific research.

Through the cluster timeline diagram (Figure 6C), we can clearly observe the developmental trajectories of different fields over time. Emerging fields such as “promising opportunities” (#14) and “MSC-derived extracellular vesicles” (#3) exhibit rapid growth, reflecting the widespread attention and investment by the scientific community in new technologies and methods. In contrast, more established fields such as “spinal cord injury pathobiology” (#9) and “neurodegenerative diseases” (#12) demonstrate stable output, indicating that research in these areas continues to progress. Additionally, fields like “rat” (#7) show a decline or stagnation in output, which may be attributed to the introduction of new technologies or a shift in academic focus.

### 3.6 Analysis of keyword

#### 3.6.1 Keyword frequency analysis

**FIGURE 7:**
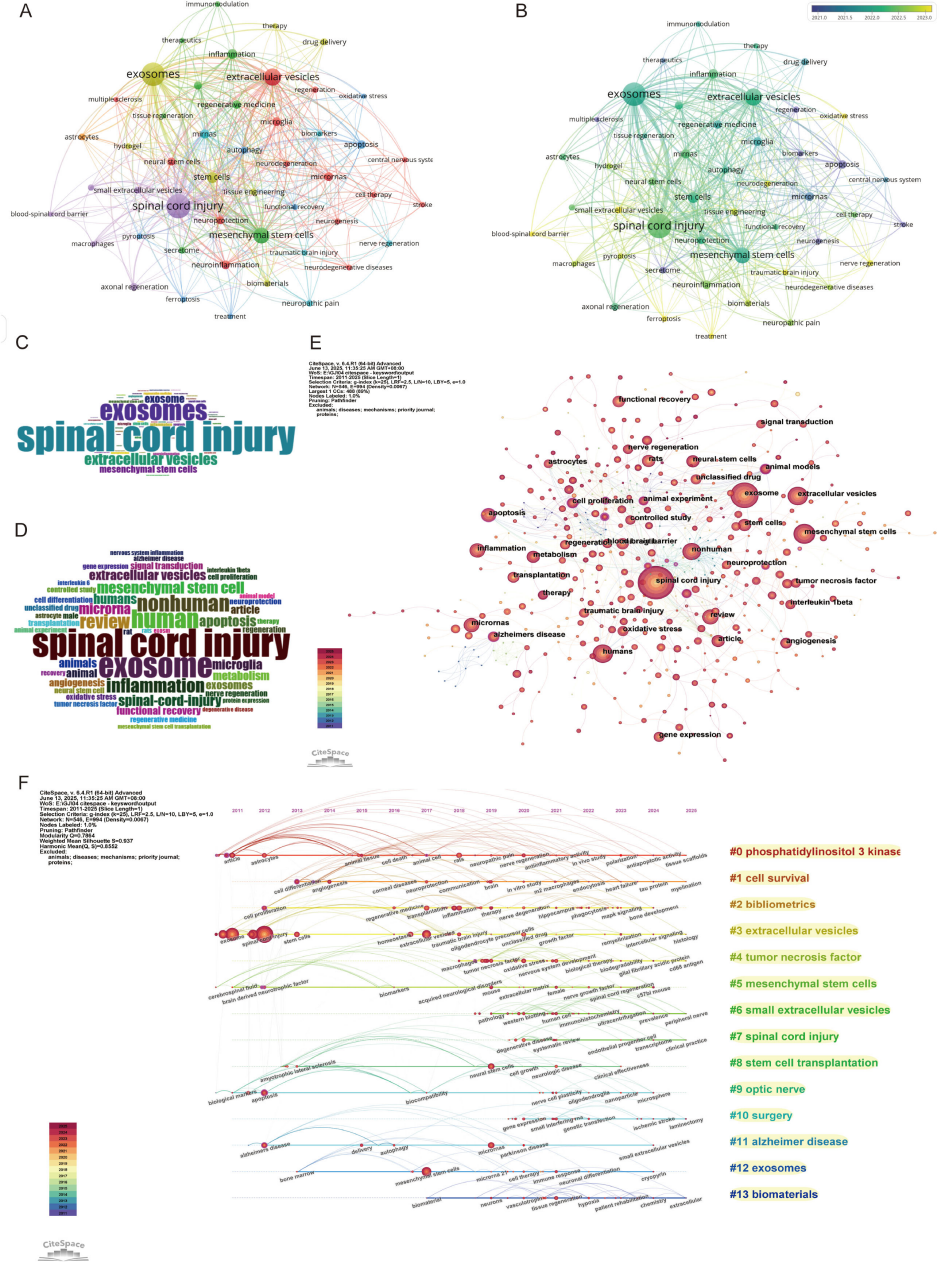
Keywords and co-citation analysis in spinal cord injury. **(A)** Keyword co-occurrence network (VOSviewer) (each node represents a keyword, and the lines between nodes represent co-occurrences. The colors of the nodes correspond to different clusters of related topics, and the size of the nodes indicates the frequency of the keywords.); **(B)** Keywords co-occurrence network with a timeline (VOSviewer) (different colors represent the average citation year of the keywords, with lighter colors indicating more recent years.); **(C)** Word cloud of author keywords (Bibliometrix) (the size of each word represents its frequency of occurrence, with larger words appearing more often in the research papers.); **(D)** Word cloud of keywords plus (Bibliometrix); **(E)** Keyword co-citation network map (CiteSpace); **(F)** Keywords clustering timeline map (CiteSpace).

Keywords represent the focus of research, and they can identify research hotspots and trends within a specific field. The bibliometrix package was used to perform statistical analysis on both Keywords Plus and Author Keywords, yielding a total of 5,579 Keywords Plus and 1,666 Author Keywords. Subsequently, VOSviewer and CiteSpace were employed to merge and analyze the keywords. The results from VOSviewer indicated that there were 1,666 Author Keywords, with 57 keywords appearing more than eight times (Figure 7A). In CiteSpace, the g-index (K = 25) was applied to select 545 keywords for subsequent analysis, aiming to include as much data as possible within the permissible range. In the keyword co-occurrence network, the number of nodes corresponds to the total number of keywords included in the analysis (Figures 7E, F). The size of the nodes reflects the frequency of the keywords’ appearance, while the connections between nodes indicate the co-occurrence of two keywords within the same paper.

Frequency analysis was conducted on Keywords Plus, Author Keywords, and all keywords, and the results are presented in the form of word clouds (Figures 7C, D). The top 10 keywords primarily focus on treatment methods for spinal cord injury, particularly research on exosomes, stem cell therapy, as well as inflammation and immune mechanisms. Other areas of research include neurobiology, cellular mechanisms, and repair processes. Among these, the keyword “spinal cord injury” appeared most frequently, followed by “exosomes,” “mesenchymal stem cells,” and “inflammation.” When analyzing the centrality, degree, and σ (Σ) values of the nodes, the terms most commonly associated with spinal cord injury and exosome research were “mesenchymal stem cells” “cell therapy” and “stem cells” (Supplementary Table 3). These terms are emphasized due to their relevance to recovery and repair mechanisms, which are closely associated with the role of exosomes in cellular communication and the healing process.

#### 3.6.2 Keyword evolution and emergence analysis

The keyword network diagram and associated data generated by VOSviewer (Figure 7B) reveal trends based on the average publication years. Keywords such as “ferroptosis,” “pyroptosis,” and “blood-spinal cord barrier” exhibit more recent average publication years around 2023, indicating that these topics represent emerging research frontiers, potentially related to novel forms of cell death mechanisms or neuroprotective strategies. In contrast, keywords like “spinal cord injury,” “exosomes,” and “mesenchymal stem cells” are concentrated around 2022, highlighting their continued prominence in current research, particularly in the fields of tissue repair and cellular therapy. On the other hand, keywords such as “multiple sclerosis” and “stroke” with average publication years around 2020, suggest that these areas of research may have reached a more mature stage or are now entering deeper phases of investigation. In terms of citation impact, keywords such as “therapeutics,” “stroke,” and “immunomodulation” demonstrate higher average citation counts, indicating that their research outcomes have substantial academic or clinical value. Additionally, topics related to “axonal regeneration” and “functional recovery,” which are closely tied to nerve repair, also show significant citation counts, reflecting the ongoing attention in the field of regenerative medicine. It is noteworthy that some emerging topics, such as “ferroptosis” and “traumatic brain injury,” despite their recent publication years, have lower average citation counts, likely due to their research being in the early stages. These topics, however, hold significant potential for future development. Regarding research intensity and connectivity, keywords like “spinal cord injury,” “exosomes,” and “mesenchymal stem cells” show high link strength and frequency of occurrence, indicating their central role within the research network and suggesting that they serve as hubs for interdisciplinary research. Furthermore, topics such as “neuroinflammation” and “neurodegeneration” are associated with multiple clusters, indicating their broad impact on research in neurological diseases.

**FIGURE 8:**
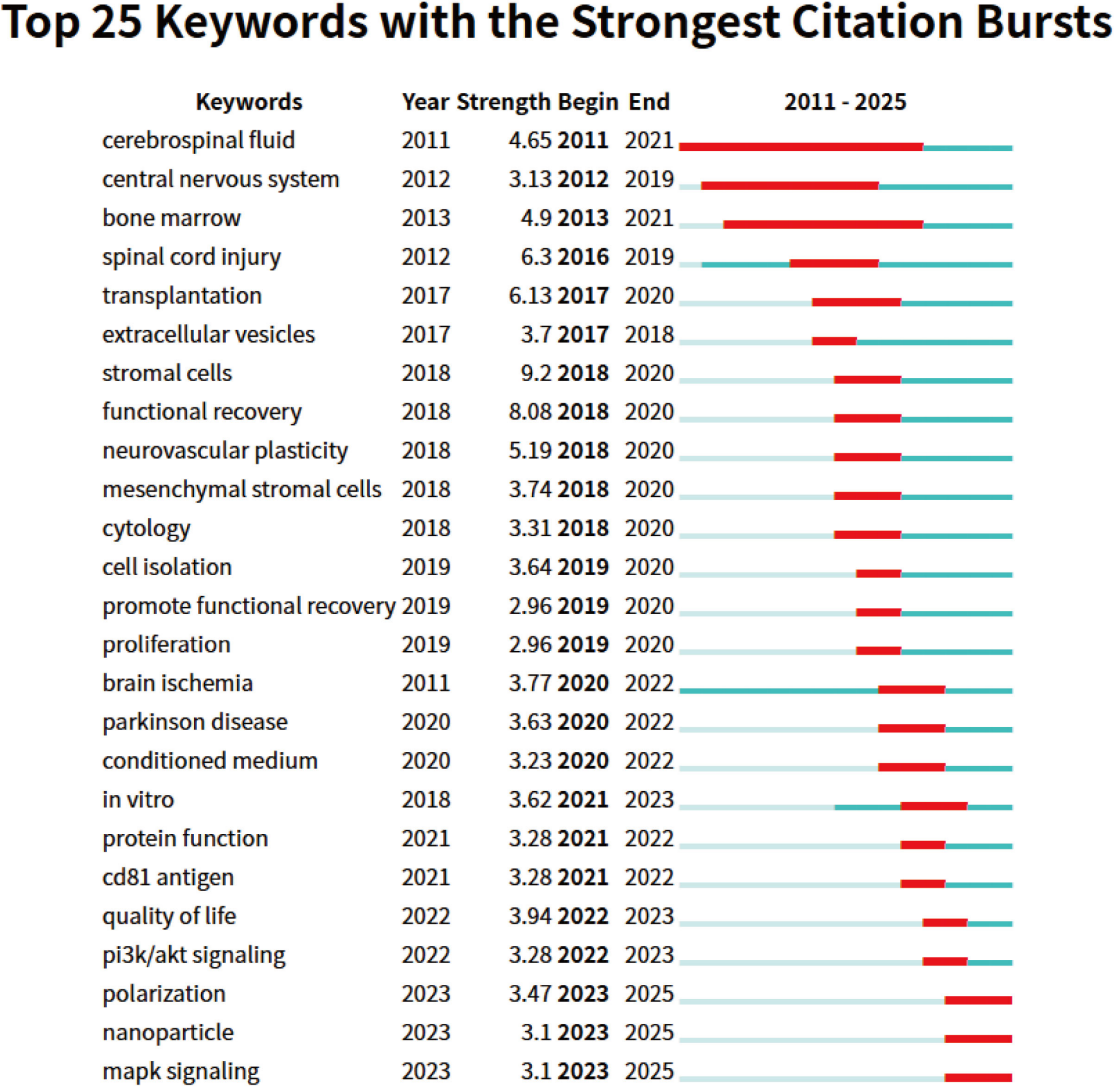
Top 25 keywords with strongest citation bursts on exosomes in spinal cord injury. The blue line indicates the timeline, and the red phase represents the outbreak period. It is based on the burst test to detect sudden changes in information like documents and keywords.

Using CiteSpace for keyword burst analysis (Figure 8), we can gain more detailed insights into research hotspots and cutting-edge developments within specific time periods, as well as predict future research trends. The citation burst data from 2011 to 2025 reveals that keywords can be categorized into three periods based on their burst times and intensities, reflecting different stages of academic development. In the early period (2011–2015), the research focus was primarily on basic medicine and neuroscience. Keywords such as “cerebrospinal fluid” and “bone marrow” exhibited high burst intensities. This period’s research mainly explored the foundational theories of neuroinjury and regenerative medicine, with particular attention on the potential of bone marrow in stem cell therapy and the role of cerebrospinal fluid in neuroprotection. Moving into the middle period (2016–2020), as technological advancements and clinical demands grew, the research direction shifted toward therapeutic applications. Keywords like “spinal cord injury” and “transplantation” showed significant bursts in 2016 and 2017, reflecting the academic community’s deepening focus on stem cell transplantation and spinal cord injury repair. At the same time, emerging research fields such as “stromal cells” and “extracellular vesicles” gained considerable attention from 2017 to 2018, highlighting the increasing importance of cell therapy and molecular mechanism studies in regenerative medicine. By the later period (2021–2025), precision medicine and cell therapy became central research areas. Keywords such as “Parkinson’s disease,” “conditioned medium,” and “protein function” saw rapid growth after 2020, indicating that the treatment of neurodegenerative diseases has become a core focus of academic research. Furthermore, with the ongoing development of nanotechnology, keywords like “nanoparticles” and “MAPK signaling” exhibited significant bursts, suggesting that nanotechnology has broad prospects in drug development and disease treatment.

## 4 Discussion

**FIGURE 9:**
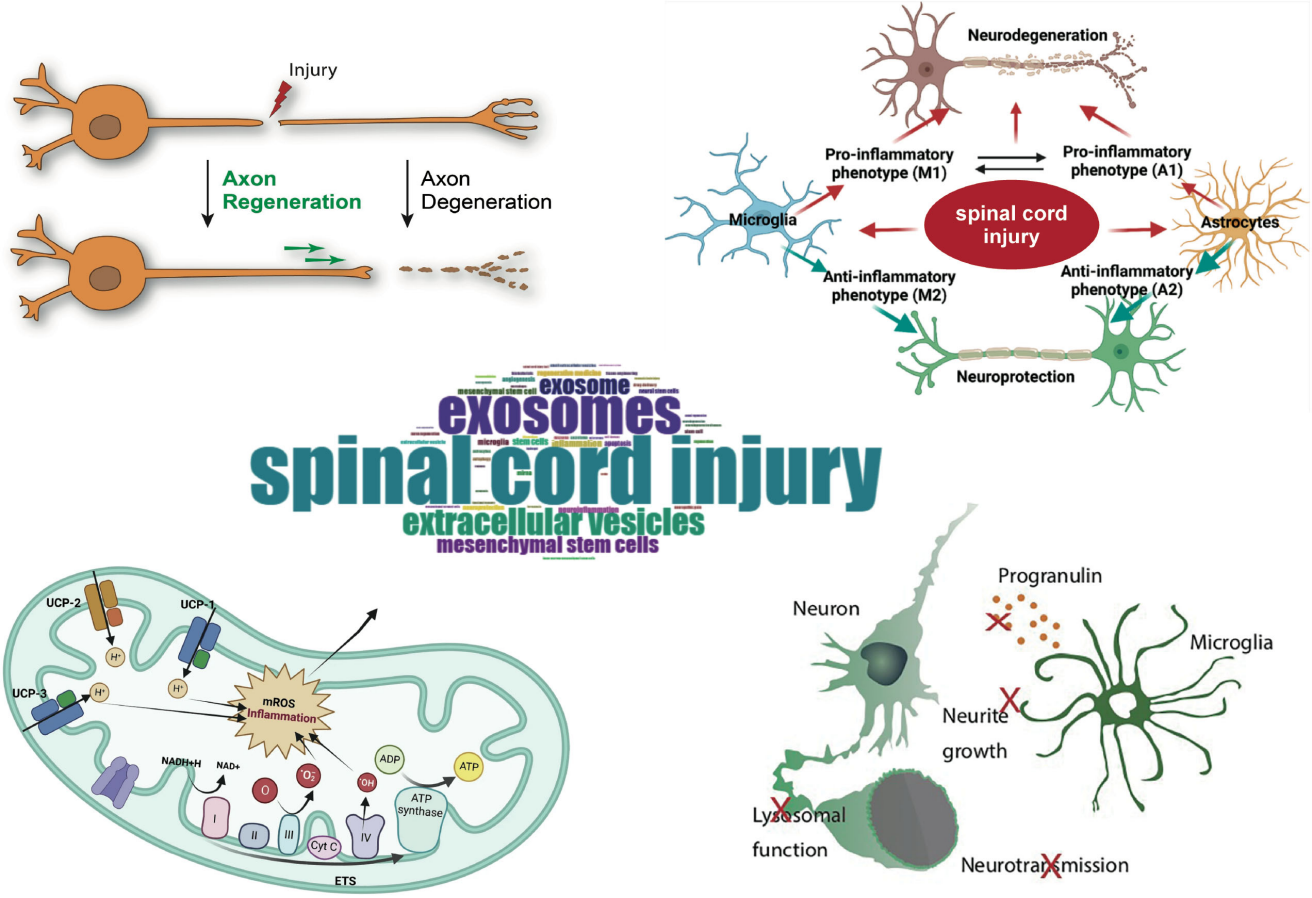
Graphical representation of spinal cord injury.

A statistical analysis was performed on 768 articles concerning the use of exosomes in SCI, which are indexed in the Web of Science and Scopus from 2011 to 2025. The objective was to assess the temporal and spatial distribution of literature in this area, contributions from authors, key publications, research hotspots, and emerging trends (Figure 9).

### 4.1 Inflammatory responses and ischemia

Spinal cord injury leads to initial damage that causes tissue destruction and triggers the activation of immune cells and inflammatory responses, further exacerbating pathological changes. Immune cells such as neutrophils, macrophages, and microglia release cytokines (such as TNF-α, IL-1β) that not only worsen the injury but also promote the role of the complement system in secondary damage. Therefore, regulating the inflammatory response has become key to treating SCI (Liu et al., 2021). The impact of ischemia (ISC) on the immune response also poses challenges for treatment; ISC exacerbates neural damage by triggering immune cascade reactions and disrupting the blood-brain barrier, and regulating the immune response has been shown to help reduce damage and accelerate recovery during the acute inflammatory phase (Gao et al., 2025; Liu et al., 2021). However, excessive activation of the immune response, particularly the overexpression of the NLRP3 inflammasome and IL-1β, may worsen secondary damage and increase treatment difficulty (Borim et al., 2025; Singh et al., 2025b). In immune regulation, macrophages (Mφ) play an important role. Mφ release pro-inflammatory factors and participate in repair through oxidative stress and the activation of the NLRP3 inflammasome. M2-type macrophages can alleviate inflammation and promote tissue regeneration, but their dual role needs to be balanced to avoid immune suppression (de Paz Linares et al., 2025; Kılıç et al., 2025; Xu et al., 2025; Yan et al., 2025). The activation of astrocytes can either exacerbate or alleviate the inflammatory response; regulating their activity can reduce pro-inflammatory cytokines, thereby mitigating damage and promoting repair (Bartak et al., 2025; Kojder et al., 2025).

### 4.2 Oxidative stress and lipid peroxidation

Oxidative stress is a significant factor in SCI, contributing to neuroinflammation and neuronal cell death. Research indicates that interventions such as gene therapy, hyperbaric oxygen therapy, and pharmacological agents like zinc ions and gallic acid can mitigate oxidative stress and facilitate recovery from SCI (Bai et al., 2025; Díaz et al., 2025; Li et al., 2025d; Liu et al., 2025c). Exosomes present a promising therapeutic avenue for alleviating oxidative stress by lowering malondialdehyde levels and boosting antioxidant activity, thereby supporting recovery from SCI (Hoseinynejad et al., 2025). Exosomes derived from bone marrow mesenchymal stem cells have demonstrated the ability to enhance oxidative stress management and mitochondrial function, offering protection to cardiomyocytes, which may also be advantageous for SCI (Zhuang Y. et al., 2025). Despite challenges related to their production and delivery, the potential of exosomes to diminish oxidative stress and assist in the repair process positions them as a promising treatment for SCI. Lipid peroxidation (LPO) is a critical contributor to secondary damage in SCI, worsening neuronal injury through mechanisms such as ferroptosis (Mai et al., 2025; She et al., 2025). Natural compounds like tanshinone IIA, flavonoids, and quercetin have been found to inhibit LPO, reduce ferroptosis, and promote healing (Li et al., 2025f; Mai et al., 2025; Xiong et al., 2025). Furthermore, ginkgo biloba extract and tetrahydropalmatine have demonstrated antioxidant properties (Li et al., 2025a; Wu Z. et al., 2025). Mesenchymal stem cell-derived exosomes may help prevent ferroptosis in SCI by modulating LPO and iron metabolism (Saadh et al., 2025). However, the role of exosomes is multifaceted, as some may worsen oxidative stress and increase LPO (Swati et al., 2025), while certain iron-rich exosomes could inhibit LPO and ferroptosis (Battaglia et al., 2025). These observations underscore the necessity for further investigation to enhance exosome-based therapies.

### 4.3 Apoptosis and necroptosis

Apoptosis and necroptosis are critical processes in SCI that significantly affect the severity of the injury and hinder recovery. Apoptosis often impedes recovery by causing cell death and worsening damage, while necroptosis, a type of programmed cell death linked to inflammation, exacerbates the injury by fostering neuroinflammation and neuronal loss. Photobiomodulation therapy has been demonstrated to decrease apoptosis and edema in the early stages of SCI, enhance motor function, and reduce the volume of injury (Campo et al., 2025). Likewise, zinc ions can mitigate apoptosis by inhibiting the TNF-α signaling pathway and diminishing immune inflammatory responses, indicating their potential therapeutic benefits (Bai et al., 2025). In SCI treatment, exosomes can modulate both apoptosis and necroptosis. Stromal cells, especially mesenchymal stem cells, show potential in promoting anti-apoptotic effects crucial for cell survival and tissue regeneration in SCI. Exosomes from adipose-derived stromal cells play a key role in inhibiting apoptosis by modulating apoptotic regulators like Bax and Bcl2, reducing the Bax/Bcl2 ratio. This modulation is vital for neural cell survival, improving recovery outcomes (Ma et al., 2025; Yao et al., 2025). However, in certain situations, particularly when loaded with chemotherapeutic agents, exosomes may also promote apoptosis by enhancing the transmission of apoptotic signals (Zhu et al., 2025). Thus, the role of exosomes in SCI treatment is multifaceted, as they can support cell survival while also potentially triggering cell death, reflecting a dual role in the recovery process of SCI (Hollanda et al., 2025). Similar to apoptosis, necroptosis can worsen neuroinflammation and neuronal damage through programmed cell death in SCI. Research has indicated that inhibiting necroptosis (e.g., using luteolin and sodium butyrate) can help reduce neuronal loss and inflammation, thereby alleviating injury (Cui et al., 2025; Liu et al., 2025e). The activation of necroptosis-related pathways, such as the ACVR1 and METTL3-NLRP3 axis, has been linked to increased neuropathic pain, suggesting that targeting these pathways may offer therapeutic opportunities (Guan et al., 2025; Zhang et al., 2025d). Furthermore, exosomes can mitigate the necroptosis process by modulating necroptosis-related pathways (for instance, by delivering miR-20a-5p that targets the TXNIP/NLRP3 pathway via M2 polarized macrophage exosomes), thereby reducing inflammation and promoting recovery from SCI (Zhang et al., 2025c).

### 4.4 Ferroptosis

Spinal cord injury is intricately linked to ferroptosis, an iron-dependent form of cell death characterized by oxidative stress and iron overload. This process significantly contributes to neuronal damage and impairs recovery following SCI (She et al., 2025). Key indicators of ferroptosis activation in SCI include elevated lipid peroxidation and the expression of ferroptotic proteins, such as ACSL4. Additionally, proteins like TRIM32 and TRIM28 play critical roles in promoting ferroptosis, which exacerbates neuronal injury and impedes motor recovery (Liu et al., 2025b; Zhou et al., 2025). The deposition of excess iron post-SCI further drives ferroptosis in microglia, contributing to sustained neuronal loss (Ouyang et al., 2025)

Several therapeutic approaches have shown promise in targeting ferroptosis to mitigate its detrimental effects on SCI. Natural flavonoids and tetramethylpyrazine (TMP) have demonstrated potential in reducing iron overload and lipid peroxidation, offering neuroprotective effects and improving motor function in SCI models (Li et al., 2025f; Tao et al., 2024). In addition, combining exosomes with deferoxamine in a hydrogel scaffold has proven effective in inhibiting ferroptosis and enhancing recovery following SCI (Zou et al., 2025). Exosomes have emerged as a promising therapeutic strategy, potentially reversing ferroptosis and promoting neuroprotection in SCI. Beyond neuronal damage, ferroptosis also contributes to the destruction of the blood-spinal cord barrier (BSCB), particularly through excessive ferroptosis in endothelial cells, further complicating SCI recovery (Wu M. et al., 2025). Nanoparticles have emerged as a significant tool in modulating ferroptosis, an iron-dependent cell death process relevant to SCI. For example, nanoparticles coated with bovine serum albumin can induce ferroptosis by releasing metal ions that catalyze the Fenton reaction, producing reactive hydroxyl radicals and enhancing therapeutic efficacy in cancer treatment (Yin et al., 2025). On the other hand, nanoparticles like Lip-1 inhibit ferroptosis by chelating iron and upregulating glutathione peroxidase 4, suggesting a protective role in neurodegenerative diseases (Zhang et al., 2025e). Additionally, cobalt nanoparticles can induce ferroptosis through lysosomal degradation and reactive oxygen species generation, showing their dual role in either promoting or inhibiting cell death depending on the context (Fan and Guo, 2025). This complexity of nanoparticle-mediated ferroptosis modulation offers potential therapeutic strategies for SCI and other neurodegenerative diseases.

### 4.5 Axonal regeneration

Promoting axonal regeneration following SCI is a crucial objective in therapy. Exosomes derived from human umbilical cord mesenchymal stem cells (UCMSCs) show significant therapeutic potential by reducing inflammation and promoting the growth of new neurons and axons when combined with biomimetic magnetoelectric hydrogels, thereby accelerating axonal regeneration and functional recovery after SCI (Liu et al., 2025a). Additionally, exosomes promote nerve repair by clearing myelin debris and enhancing axonal myelination (Li et al., 2026; Zamanian et al., 2025). In peripheral nerve injuries, exosomes also demonstrate their potential to promote axonal regeneration, myelin repair, muscle protection, and vascularization (Aldali et al., 2025; Chang et al., 2025). Neural stem cells (NSCs) play an important role in axonal regeneration after SCI. When NSCs are combined with mesenchymal progenitor cells, they can improve the survival rate of NSCs and promote their differentiation into oligodendrocytes, further enhancing axonal regeneration (White et al., 2025). Furthermore, the combined treatment of UCMSCs and their secretions significantly improved motor function and language abilities in patients with traumatic axonal injury (Faried et al., 2025). VEGF promotes vascular and axonal regeneration after spinal cord injury through exosomes (Yun et al., 2025). Basic fibroblast growth factor (bFGF) combined with delivery systems such as hydrogels significantly enhances axonal regeneration and neural functional recovery (Li et al., 2023; Zhu et al., 2024). Recombinant fibroblast growth factor (rFGF4) further promotes nerve repair by regulating the polarization of microglia and macrophages to the reparative M2 subtype (Li et al., 2024). These studies support the potential of interventions in the treatment of SCI and provide new directions for future therapeutic strategies.

### 4.6 Neuronal regeneration and synaptic plasticity

Neuronal regeneration and synaptic plasticity are key to recovery from SCI. Research indicates that transcutaneous auricular vagus nerve stimulation enhances nerve regeneration by promoting myelin repair and axonal remodeling, which is associated with improvements in synaptic plasticity. This is evidenced by restored synaptic ultrastructure and increased functional imaging metrics, both of which correlate with neurological recovery (Zhang et al., 2025b). Additionally, inhibiting the NgR1 factor can increase synaptic plasticity and improve neuronal regeneration (Zhang et al., 2025a). These findings suggest that neuronal regeneration is closely related to synaptic plasticity, and combined treatment of both may aid in the recovery from spinal cord injury. Exosomes have been shown to promote neuronal regeneration and support synaptic plasticity. Exosomes derived from BMSCs can reduce apoptosis, inhibit inflammation, promote axonal growth, and improve motor function (Liu et al., 2025d). When used in conjunction with hydrogels, BMSCs further promote neuronal proliferation and differentiation, enhancing functional recovery (Sun et al., 2025). BMSCs exosomes rich in miR-219-5p and the miR-17-92 cluster can reduce neuronal apoptosis, inhibit ferroptosis, and promote neurological recovery (Dong et al., 2024; Wang et al., 2024). In addition, the combined use of BMSCs transplantation and double leaf glycosides can accelerate neuronal differentiation and improve functional recovery (Chen et al., 2024). Studies have shown that exosomes derived from antler bud progenitor cells are more effective in SCI repair than exosomes derived from BMSCs (Yang S. et al., 2025). Research on amniotic membrane transplantation and mesenchymal progenitor cell transplantation indicates that these exosomes can promote nerve regeneration, support the survival of neural stem cells, and facilitate oligodendrocyte differentiation (Parmar et al., 2025; White et al., 2025).

### 4.7 Drug delivery systems

Drug delivery systems (DDS) are essential for enhancing the treatment of SCI by improving the administration and effectiveness of therapeutic agents. Intrathecal targeted drug delivery systems have been found to significantly decrease the analgesic needs of patients with cervical SCI, offering a viable alternative for those who do not respond to standard therapies (Yoo et al., 2025). Exosomes, as an innovative DDS, can efficiently transport bioactive molecules that encourage anti-inflammatory and neurogenic processes, addressing the challenges of regeneration in SCI (Mao et al., 2025). However, the application of intrathecal DDS may also result in serious complications, such as spinal cord injury due to catheter tip granulomas, underscoring the importance of careful monitoring (Mo et al., 2025). Advanced DDS, such as bigels, provide mechanical support and localized therapeutic delivery, showing potential in enhancing neurotherapeutic applications for SCI (Moswatsi et al., 2025). The development of dual DDS, like Nit-MNs, has shown promise in facilitating SCI repair by improving microglial homeostasis and restoring neural function (He et al., 2025). Additionally, combining nanoparticle drug delivery systems with Plexin B2 targeted therapy can improve neural healing following SCI (Wang Q. et al., 2025). A polymer-based nanoparticle DDS, PEG-PCL-ACPP, has been created to enhance targeted delivery and sustained release of glibenclamide at the SCI site, demonstrating significant anti-inflammatory and neuroprotective effects (Zhuang Z. et al., 2025). Moreover, the localized delivery of extracellular vesicles derived from mesenchymal stem cells via hydrogel systems has notably improved recovery outcomes in SCI models (Cao et al., 2025). In summary, research on various DDS (including nanoparticles, hydrogels, and collagen scaffolds) is crucial for improving the effectiveness of SCI treatment, as the current treatment outcomes are still unsatisfactory (Li et al., 2025c).

### 4.8 Research trends and future directions in SCI therapeutic technologies

Recent advancements in SCI treatment include stem cell therapy, which has improved neuron survival and functional recovery through neuroectodermal stem cells (NESCs) and MPCs. Autologous bone marrow mesenchymal stem cell transplantation has also shown efficacy in enhancing motor and sensory functions in SCI patients (Alastra et al., 2025; Bellák et al., 2025; White et al., 2025). Hydrogel technology, with its excellent biocompatibility and conductivity, has demonstrated significant potential in SCI treatment. Piezoelectric hydrogels promote neural stem cell differentiation and motor function recovery, and combining hydrogels with genetically engineered cells enhances the therapeutic effect (Han et al., 2025; Shi et al., 2025; Zhao et al., 2025). Exosomes can promote neuronal regeneration, reduce inflammation and apoptosis, and when combined with hydrogels, further enhance treatment effects (Chen et al., 2025; Liu et al., 2025d). Bone material technology, particularly when combined with bone marrow stem cells and growth factors, shows promise in improving functional recovery and bone regeneration, though challenges remain in material standardization and production (Jagadale et al., 2025; John et al., 2025), Gene therapy delivers anti-inflammatory cytokines and regulates autophagy to promote neural function recovery (Choi et al., 2025; Ding et al., 2025). DDS, such as exosomes as carriers, are emerging as new methods for SCI treatment due to their targeting capabilities and nerve regeneration properties (Mao et al., 2025; Ullrich et al., 2025). These technologies provide promising prospects for SCI treatment and help optimize future clinical strategies. With advancements in regenerative medicine, biomaterials, and genetic engineering, SCI treatments are becoming more precise, personalized, and systematic. The development of intelligent biological scaffolds will enhance the directional release of active factors and maintain cell functions (You et al., 2023). Additionally, the integration of digital technologies like AI and big data will accelerate the personalization of treatment decisions (You et al., 2023). Despite challenges like ethical regulation and cell source safety, interdisciplinary integration suggests that SCI treatment will shift from “replacement repair” to “functional regeneration” (Raji et al., 2025).

### 4.9 Challenges and prospects

Exosomes have shown great potential in the treatment of SCI, but there are several challenges in their clinical translation, primarily related to the standardization of production, treatment safety, and the complexity of their mechanisms of action. The lack of standardized protocols for the production and management of exosomes has led to inconsistencies in clinical applications, limiting their widespread use (Lin et al., 2025). Additionally, the compositional variability and complex mechanisms of action of exosomes, particularly the involvement of specific miRNAs such as miR-5121 and miR-24-3p, make their therapeutic effects and safety more difficult to predict (Li et al., 2025e; Wang X. et al., 2025). Moreover, exosomes induced by SCI may possess pro-inflammatory characteristics, which could exacerbate tissue damage and increase inflammation, highlighting the need for strategies to mitigate these adverse effects (Fang Y. et al., 2025). Currently, there is only 1 registered clinical trial, and large-scale clinical application still needs to break through the production standardization barrier (Supplementary Figure 1).

Nevertheless, the prospects of exosome therapy for SCI remain highly promising. With advances in gene engineering, nanotechnology, and other fields, exosomes are expected to enable more precise and personalized treatments. Exosomes derived from stem cells have shown multiple beneficial effects, particularly in enhancing neural regeneration, reducing inflammation, and stabilizing the blood-spinal cord barrier, offering significant therapeutic potential (Lin et al., 2025). For instance, exosomes derived from A2 astrocytes have been shown to play a key role in promoting neurofunctional recovery and repairing the blood-spinal cord barrier in mice through the miR-5121-mediated AKT2/mTOR/p70S6K pathway (Wang X. et al., 2025). Additionally, the combination of umbilical mesenchymal stem cell-derived exosomes and neural stem cell fibers has effectively improved motor function recovery and reduced inflammation (Li et al., 2025b).

Clinically, exosomes also show potential in drug delivery and axon myelination. Intrathecal administration of exosomes purified from human plasma has been demonstrated to improve axonal myelination and reduce cavity size in rats following spinal cord injury, further indicating their beneficial role in recovery (Zamanian et al., 2025). Moreover, exosomes derived from bone marrow mesenchymal stem cells have been found to alleviate spinal cord injury by reducing cell apoptosis and inflammation, with miR-24-3p playing a crucial role in this protective effect (Li et al., 2025e).

Looking ahead, the prospects for exosome therapy remain extremely promising. As technology continues to evolve, exosomes are expected to be combined with other therapeutic approaches, such as gene therapy and stem cell therapies, to further enhance efficacy and overcome the limitations of single-treatment methods. With improvements in production processes and standardization, exosome therapy is poised to become an effective treatment for spinal cord injury in the future.

## 5 Limitations

Although this study conducted a comprehensive bibliometric analysis of exosome-based therapies for spinal cord injury from 2011 to 2025, several limitations must be acknowledged. First, the analysis relied solely on the Web of Science and Scopus databases, excluding other sources and gray literature, which may have led to the omission of important research. Second, citation counts as a measure of impact may be biased, as they are influenced by journal impact factors, potentially overlooking newer or innovative studies. Additionally, the study is dominated by research from China, which may not fully represent global contributions, particularly from regions with lower research output. The time frame of 2011–2025 also limits the capture of earlier foundational work or recent emerging trends. Due to the lack of a national-level H-index calculation tool, total citations/citations per capita was used as a proxy in this study, and future research suggests the development of an algorithm for this purpose. Despite using bibliometric tools like CiteSpace, VOSviewer, and R-bibliometric, their analytical scope is limited, particularly in capturing interdisciplinary complexities. Furthermore, while the study identifies trends in exosome therapy for spinal cord injury, it lacks an in-depth exploration of clinical applications, which are influenced by patient variability, treatment protocols, and long-term outcomes. Lastly, the dataset was intentionally focused on English-language publications to ensure consistency and comparability. While this approach facilitated a unified analysis, it may have underrepresented research contributions from non-English-speaking regions, potentially excluding valuable insights from global research and affecting the completeness of our findings. Future studies could consider expanding the scope of the data to include non-English publications to provide a more comprehensive representation of global research.

## 6 Conclusion

Our study underscores the growing interest in exosome-based therapies for SCI, focusing on their preclinical potential in promoting neuroprotection, axonal regeneration, and modulating inflammation. While promising, further research is needed to investigate the integration of exosome therapies with advanced drug delivery systems and regenerative medicine to optimize SCI treatment and personalize recovery strategies in clinical settings.
