# Supplementary material for: Emerging regenerative strategies for spinal cord injury: exosome-derived mechanisms and therapeutic insights
Source: Front Neurosci. 2025 Aug 25;19:1652196. doi: 10.3389/fnins.2025.1652196 (PMC12415016; doi:10.3389/fnins.2025.1652196)
Supplement: Supplementary file 1 [file Data_Sheet_1.docx]

**Supplement** **Figure**

**Supplement** **Figure S1**. PubMed Search Strategy for Spinal Cord Injury and Exosomes Research

**Supplement Figure S1**. PubMed Search Strategy for Spinal Cord Injury and Exosomes Research
