# Supplementary material for: Emerging regenerative strategies for spinal cord injury: exosome-derived mechanisms and therapeutic insights
Source: Front Neurosci. 2025 Aug 25;19:1652196. doi: 10.3389/fnins.2025.1652196 (PMC12415016; doi:10.3389/fnins.2025.1652196)
Supplement: Supplementary file 2 [file Supplementary_file_1.docx]

**Exosomes in Spinal Cord Injury Research(1996–2025): Scientometric Analysis of 30 Years Research**

**Running title: Exosomes in Spinal Cord Injury**

**Supplemental Tables**

**Supplementary Table 1.** Bibliometric Overview of Exosomes in Spinal Cord Injury (2011–2025)

**Supplementary Table 2.** Most Relevant Countries by Corresponding Author Contributions (2011–2025)

**Supplementary Table S3**. Top 10 Keywords based on CiteSpace 's degree, Centrality, Sigma

**Supplementary Table 1.** Bibliometric Overview of Exosomes in Spinal Cord Injury (2011–2025)

| **Description** | **Results** |
| --- | --- |
| MAIN INFORMATION ABOUT DATA |  |
| Timespan | 2011:2025 |
| Sources (Journals, Books, etc) | 308 |
| Documents | 768 |
| Annual Growth Rate % | 28.51 |
| Document Average Age | 2.92 |
| Average citations per doc | 33.74 |
| References | 53244 |
| DOCUMENT CONTENTS |  |
| Keywords Plus (ID) | 5579 |
| Author's Keywords (DE) | 1666 |
| AUTHORS |  |
| Authors | 3586 |
| Authors of single-authored docs | 11 |
| AUTHORS COLLABORATION |  |
| Single-authored docs | 12 |
| Co-Authors per Doc | 7.22 |
| International co-authorships % | 8.073 |
| DOCUMENT TYPES |  |
| article | 603 |
| review | 165 |

**Supplementary Table 2.** Most Relevant Countries by Corresponding Author Contributions (2011–2025)

| **Rank** | **Country** | **Articles** | **Articles %** | **SCP** | **MCP** | **MCP %** |
| --- | --- | --- | --- | --- | --- | --- |
| 1 | CHINA | 334 | 43.5 | 313 | 21 | 6.3 |
| 2 | USA | 31 | 4 | 23 | 8 | 25.8 |
| 3 | IRAN | 26 | 3.4 | 18 | 8 | 30.8 |
| 4 | KOREA | 11 | 1.4 | 10 | 1 | 9.1 |
| 5 | INDIA | 7 | 0.9 | 4 | 3 | 42.9 |
| 6 | AUSTRIA | 6 | 0.8 | 3 | 3 | 50 |
| 7 | ITALY | 6 | 0.8 | 4 | 2 | 33.3 |
| 8 | JAPAN | 6 | 0.8 | 5 | 1 | 16.7 |
| 9 | RUSSIA | 5 | 0.7 | 4 | 1 | 20 |
| 10 | UNITED KINGDOM | 5 | 0.7 | 3 | 2 | 40 |
| 11 | GERMANY | 4 | 0.5 | 3 | 1 | 25 |
| 12 | AUSTRALIA | 3 | 0.4 | 1 | 2 | 66.7 |
| 13 | EGYPT | 3 | 0.4 | 2 | 1 | 33.3 |
| 14 | ISRAEL | 3 | 0.4 | 1 | 2 | 66.7 |
| 15 | MALAYSIA | 3 | 0.4 | 3 | 0 | 0 |

**Supplementary Table S3**. Top 10 Keywords based on CiteSpace 's degree, Centrality

| **Rank** | **Degree** | **Node Name** | **Centrality** | **Node Name** |
| --- | --- | --- | --- | --- |
| 1 | 0.56 | mesenchymal stromal cells | 49 | spinal cord injury |
| 2 | 0.54 | cell therapy | 31 | extracellular vesicles |
| 3 | 0.53 | spinal cord injury | 27 | mesenchymal stem cells |
| 4 | 0.51 | animal models | 17 | stem cells |
| 5 | 0.5 | conditioned medium | 16 | neural stem cells |
| 6 | 0.47 | cell-based therapy | 15 | mesenchymal stromal cells |
| 7 | 0.39 | cell transplantation | 14 | cell therapy |
| 8 | 0.35 | cell-free therapy | 11 | neurodegenerative diseases |
| 9 | 0.29 | mesenchymal stem cells | 11 | blood-brain barrier |
| 10 | 0.28 | stem cells | 10 | good manufacturing practice |
